# Pylorus drainage procedures in thoracoabdominal esophagectomy – a single-center experience and review of the literature

**DOI:** 10.1186/s12893-018-0347-x

**Published:** 2018-03-01

**Authors:** Stefan Fritz, Katharina Feilhauer, André Schaudt, Hansjörg Killguss, Eduard Esianu, René Hennig, Jörg Köninger

**Affiliations:** Department of General, Visceral, Thoracic and Transplantation Surgery, Katharinenhospital Stuttgart, Kriegsbergstraße 60, 70174 Stuttgart, Germany

**Keywords:** Thoracoabdominal esophagectomy, Pylorotomy, Pyloroplasty, Pylorus drainage, Delayed gastric emptying

## Abstract

**Background:**

Pylorotomy and pyloroplasty in thoracoabdominal esophagectomy are routinely performed in many high-volume centers to prevent delayed gastric emptying (DGE) due to truncal vagotomy. Currently, controversy remains regarding the need for these practices. The present study aimed to determine the value and role of pyloric drainage procedures in esophagectomy with gastric replacement.

**Methods:**

A retrospective review of prospectively collected data was performed for all consecutive patients who underwent thoracoabdominal resection of the esophagus between January 2009 and December 2016 at the Katharinenhospital in Stuttgart, Germany. Clinicopathologic features and surgical outcomes were evaluated with a focus on postoperative nutrition and gastric emptying.

**Results:**

The study group included 170 patients who underwent thoracoabdominal esophageal resection with a gastric conduit using the Ivor Lewis approach. The median age of the patients was 64 years. Most patients were male (81%), and most suffered from adenocarcinoma of the esophagus (75%). The median hospital stay was 20 days, and the 30-day hospital death rate was 2.9%. According to the department standard, pylorotomy, pyloroplasty, or other pyloric drainage procedures were not performed in any of the patients. Overall, 28/170 patients showed clinical signs of DGE (16.5%).

**Conclusions:**

In the literature, the rate of DGE after thoracoabdominal esophagectomy is reported to be approximately 15%, even with the use of pyloric drainage procedures. This rate is comparable to that reported in the present series in which no pyloric drainage procedures were performed. Therefore, we believe that pyloric drainage procedures may be unwarranted in thoracoabdominal esophagectomy. However, future randomized trials are needed to ultimately confirm this supposition.

## Background

Thoracoabdominal esophagectomy for esophageal cancer has been associated with high rates of morbidity and mortality in the past. Until the 1980s, postoperative in-hospital death rates were reported to range around 30% [1, 2]. Due to significant improvements in surgery, anesthesiology, and intensive care management, a reduction in mortality to less than 10% has been achieved in the last 10–20 years [3–5]. Along with improvements in peri- and postoperative outcomes, surgical techniques have also been evaluated with regard to non-life-threatening postoperative complications and quality of life.

Delayed gastric emptying (DGE) is a frequent functional disorder of the pylorus following esophagectomy with a gastric conduit. Depending on the definition and the surgical technique performed, clinical symptoms during the postoperative course reportedly occur in 10% to 50% of patients [6, 7]. Gastric outlet obstruction results from truncal vagotomy and is thought to be associated with an increased incidence of postoperative complications, including aspiration with subsequent pneumonia and anastomotic leaks. Consequently, DGE is reported to lead to decreased patient satisfaction and a prolonged hospital stay [8–10].

Although gastric tube reconstruction, rather than reconstruction of the entire stomach, has been sufficiently demonstrated to be associated with a significantly reduced risk of DGE [11], superior quality-of-life scores during the first postoperative year, and less reflux esophagitis, controversy surrounds the need for pyloric drainage procedures [9, 12]. Based on historical experience with truncal bilateral vagotomies during peptic ulcer surgery, pyloric drainage procedures were routinely performed in many high-volume centers to prevent DGE. The choice of procedure, including pylorotomy, pyloroplasty, finger fracture, or botulinum toxin injection, mainly depended on the surgeon’s preference. Recent series have questioned the benefit of these procedures [13]. Currently, ongoing controversy remains concerning the need for pyloric drainage procedures following esophageal substitution with gastric interposition [8, 14, 15].

Limited data are available on short-term postoperative outcomes regarding DGE following esophagectomy with gastric pull-up. Moreover, most reports are based on series that include patients who underwent surgery in the 1980s and 1990s or even earlier [16–19]. At that time, mortality rates were two- to three-times higher compared to those in more recently published series in high-volume centers. Furthermore, the use of pyloric drainage procedures was based on different surgical schools and not on evidence or randomized clinical trials. In recent years, the rate of laparoscopic esophagectomies has continuously increased. Certainly, pylorus drainage procedures can be performed laparoscopically. However, this technique is relatively sophisticated and may be related to morbidity. Especially for the laparoscopic approach, it is important to be sure whether pylorus drainage procedures will be beneficial compared to no intervention. Therefore, the present study aimed to determine the value and role of pylorus drainage procedures in esophagectomy.

## Methods

### Patients

All consecutive patients who underwent thoracoabdominal esophagectomy at the Department of General, Visceral and Transplantation Surgery of the Katharinenhospital in Stuttgart, Germany between January 2009 and December 2016 were prospectively evaluated from a database. The study was approved by the institutional review board of the Klinikum Stuttgart and is in compliance with the Declaration of Helsinki. Clinicopathologic features including age, sex, body mass index, tumor location and stage, and nutrition management were assessed. Clinical courses and outcomes were evaluated with a focus on postoperative nutrition and gastric emptying difficulties. Among other variables, the rate of gastric outlet obstruction symptoms, the median postoperative day of return to a normal full diet, and postoperative surgical and non-surgical complications were assessed. Moreover, clinical management of gastric outlet obstruction was investigated with regard to success rates and outcomes.

### Preoperative work-up

All patients included in the study had undergone preoperative thin-sliced radiological imaging using computed tomography or magnetic resonance imaging (MRI). All images were accessed and viewed using the PACS online imaging system of the Katharinenhospital in Stuttgart, Germany (GE Healthcare, Barrington, USA). For preoperative staging, all patients underwent endoscopic ultrasonography examination (EUS) to assess tumor size, infiltration depth and enlargement of lymph nodes. All patients were presented at an interdisciplinary tumor board for therapeutic management planning. According to preoperative tumor staging, patients received neoadjuvant chemotherapy or radiochemotherapy as indicated.

### Surgical procedure

**Fig. 1:**
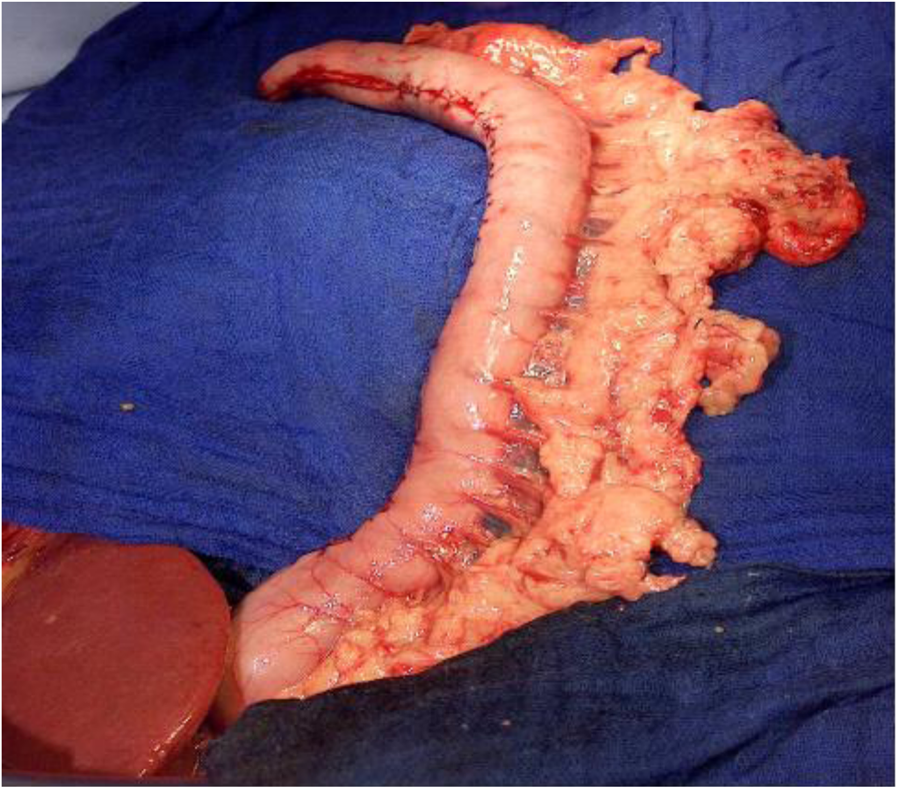
Gastric conduit for esophageal replacement All patients received thoracoabdominal esophagectomy with gastric pull-up. The gastric conduit was small, with a diameter of 3–5 cm. None of the patients received any pyloric drainage procedure such as pylorotomy, pyloroplasty, pylorus buginage or botulinum toxin injection.

All study patients except for three underwent open Ivor Lewis thoracoabdominal esophagectomy. In brief, an upper abdominal incision and a right-sided posterolateral thoracotomy were applied for surgical access. The procedure was mainly performed on tumors of the middle- and lower-third of the esophagus. In cases of uncertain resectability or lesions suspected of metastasis in the liver or lung, an exploratory laparotomy and/or thoracotomy was performed before surgical esophageal resection. Patients who did not undergo curative resection were excluded from the study. Only patients with the stomach as the site of esophageal replacement were included in the present study. Patients with colonic or jejunal interposition were excluded. The gastric conduit for esophageal replacement (Fig. 1) was brought up to the thorax using a posterior mediastinal route. Three patients underwent laparoscopic mobilization of the stomach and open thoracotomy for gastric pull-up and anastomosis.

### Postoperative management

Patients were routinely transferred to an intensive care unit postoperatively for three days, followed by another three days in an intermediate care unit with cardiopulmonary monitoring. The gastric tube was removed on postoperative day one in all patients regardless of the amount of reflux. On day one, patients were allowed to drink clear liquids ad libitum. Return to regular diet was routinely started on postoperative day four. Abdominal drainages were removed on day two and thoracic drainage according to the amount and quality of fluid production.

## Results

### Patient characteristics

A total of 170 consecutive patients who underwent thoracoabdominal resection of the esophagus with gastric replacement were included in the study (Table 1). The entire group included 137 men (80.6%) and 33 women (19.4%), with a median age of 64 years (range: 39–87 years). Most of the patients were male (80.6%). The mean body mass index of all patients was 26 kg/m^2^ (range: 16.3–41.8 kg/m^2^), with body weight ranging from 48 to 131 kg (mean: 78.2 kg).

**Table 1:** Demographics and comorbidities of the patients (*n* = 170)

Feature		Number	Percent
Age (years)	Median: 64		
	Range: 39–87		
Sex	Male	137	80.6
	Female	33	19.4
Body mass index (kg/m^2^)	Mean: 26.7		
	Range: 16.3–41.8		
	≤18.5	7	4.1
	> 18.5 and ≤25.0	73	42.9
	> 25.0 and ≤30.0	65	38.3
	> 30.0 and ≤35.0	17	10.0
	> 35.0	8	4.7
Body weight (kg)	Mean: 78.2		
	Range: 48–131		
Smoking history	Current smoker	66	38.8
	Former smoker	22	13.0
	Never	82	48.2
Alcohol history	Current alcohol abuse	35	20.6
	Former alcohol abuse	12	7.1
	Never	123	72.3
Comorbidity	Cardiovascular (hypertension excluded)	51	30.0
	Pulmonary	27	15.9
	Renal	12	7.1
	Diabetes mellitus	30	17.6
ASA risk classification	I	2	1.2
	II	60	35.3
	III	108	63.5
	IV	0	0

Most of the patients (128/170) suffered from adenocarcinoma of the esophagus (75.3%). Thirty-six patients suffered from squamous cell carcinoma (21.2%). Three patients had neuroendocrine tumors of the esophagus (1.8%), and one patient suffered from a gastrointestinal stroma tumor (GIST). Two patients underwent surgery for non-neoplastic disease, including a 73-year-old male with peptic stenosis and a 72-year-old patient who suffered from distal scarred stenosis following iatrogenic perforation during endoscopic dilation for achalasia (Table 2).

**Table 2:** Disease-specific and oncological characteristics

Feature		Number	Percent
Histological type of disease	Adenocarcinoma	128	75.2
	Squamous cell carcinoma	36	21.2
	Neuroendocrine tumor (NET)	3	1.8
	Other	3	1.8
Tumor location	Upper-third (0–18 cm)	1	0.6
	Middle-third (19–29 cm)	32	18.8
	Lower-third (30–45 cm)	137	80.6
Clinical T-stage at diagnosis	cT1	5	2.9
	cT2	38	22.4
	cT3	90	52.9
	cT4	35	20.6
	Not determined	2	1.2
Clinical N-stage at diagnosis	cN0	50	29.4
	cN+	118	69.4
	Not determined	2	1.2
Neoadjuvant therapy	Chemo- or radiochemotherapy	111	65.3

Excluding arterial hypertension, 51/170 patients (30.0%) had cardiovascular comorbidities such as coronary heart disease with status post cardiac infarction or bypass surgery. According to the American Society of Anesthesiologists (ASA) classification, 108/170 patients were categorized as ASA III (63.5%), and 60 were categorized as ASA II (35.3%).

The indication for surgical resection and the histological characteristics and locations of the tumors are listed in Table 2. Most patients (118/170) had lymph node-positive disease (69.4%) and received neoadjuvant chemo- or radiochemotherapy (65.3%).

### Surgical resection

All patients underwent thoracoabdominal esophageal resection using the Lewis-Tanner approach as described in the Patients and Methods section. According to the department standard, pyloric drainage procedures such as pylorotomy, pyloroplasty, pylorus buginage or botulinum toxin injection were not applied in any of the patients. The mean operative time was 256 min (range: 117–386 min).

### Postoperative management

In most patients (83%), the nasogastric tube was removed on postoperative day one or two. Clinical signs of gastric outlet obstruction were observed in 28/170 patients (16.5%). DGE was primarily treated conservatively with re-insertion of a gastric tube and prokinetic drugs. In 5 of 170 patients (2.9%), DGE was severe and persistent and required intervention. In these cases, endoscopic dilation of the stenotic region was performed (Table 3). In all five patients, the intervention was successful. The median return to a regular diet was satisfactory at six days postoperatively.

**Table 3:** Operative procedure and postoperative outcomes

Feature		Number	Percent
Operative time (minutes)	Mean: 256		
	Range: 117–386		
Surgical complications	Anastomotic leak	41	24.1
	Surgical site infection	29	17.1
	Delayed gastric emptying (DGE)	28	16.5
	Delayed gastric emptying (DGE) requiring intervention	5	2.9
	Chylothorax	8	4.7
	Postoperative bleeding	8	4.7
Non-surgical complications	Pneumonia	46	27.1
	Pleural effusion	42	24.7
	Cardiovascular complications	11	6.5
	Postoperative delirium	11	6.5
	Renal failure	5	2.9
Postoperative endoscopy	Not required	92	54.1
	Diagnostic endoscopy	78	45.9
	Stenting/endosponge	36	21.2
	Balloon dilation	5	2.9
Surgical re-intervention	Total	18	10.6
	Re-operations for early anastomotic leak	7	4.1
	Ligature of the thoracic duct	5	2.9
	Postoperative bleeding	3	1.8
	Other	3	1.8
Length of hospital stay (days)	Median: 20		
	Range: 8–112		
30-day mortality		5	2.9

### Surgical outcomes

The median hospital stay was 20 days (range: 8–112 days), including a mean of six days in the intensive care unit. The 30-day hospital death rate was 2.9%. Postoperative endoscopy was performed in 45.9% of the cases. Implantation of a stent or endosponge was performed in 21.2% of the cases, and endoscopic balloon dilation was applied in 2.9% of the cases. Anastomotic leakage was observed in 24.1% of the cases. However, most leakages (36/41) were sufficiently treated with endoscopic stenting or endosponge therapy (87.8%). Re-operation for anastomotic leakage was required in 7/170 patients (4.1%). Other reasons for re-operation were chylous fistula (2.9%), postoperative bleeding (1.8%), and ischemia of the gastric conduit (1.2%). Overall, surgical re-intervention was required in 18/170 cases (10.6%).

The rates of postoperative non-surgical complications are listed in Table 3. Generally, the most frequent non-surgical complications were pneumonia (27.1%) and pleural effusion (24.7%).

### Pathology

Of all 170 resected patients, 168 had an oncological diagnosis (98.8%). One patient suffered from peptic stenosis and another from stenosis following iatrogenic injury during endoscopy. Most patients had histological T3 or T4 tumors (49.7%), 35.8% of the patients had a T1 or T2 tumor, and 14.5% had ypT0 or ypTis tumors. Overall, 50.6% of the patients with an oncological diagnosis had lymph node-positive disease, and 49.4% had lymph node-negative findings on final histopathology.

## Discussion

DGE is one of the major causes of severe aspiration pneumonia, which is associated with a poor early postoperative outcome following esophagectomy with gastric replacement [20]. In the past, different surgical techniques, such as pyloromyotomy, pyloroplasty, or pylorus buginage, were implemented to reduce the incidence of gastric outlet obstruction [14].

Currently, the value of these pyloric drainage procedures remains controversial [9]. The potential advantage of these procedures is possible prevention of DGE [21]. Some authors argue that by reducing gastric outlet obstruction, the incidence of aspiration pneumonia may decrease, potentially improving early postoperative outcomes [17]. Others argue that only a minority of patients show signs of DGE. Furthermore, pyloric drainage procedures may predispose patients to dumping and duodenal reflux, which could impede late postoperative functional outcomes [14, 20, 22, 23].

In 1991, Fok et al. published a prospective randomized study on 200 patients who underwent esophagectomy with gastric replacement [17]. Based on their findings, the authors recommended pyloroplasty for patients in whom the entire stomach was used for reconstruction after esophagectomy. These results are not comparable to those of more recent studies because most centers today do not use the entire stomach for esophageal replacement but only a small conduit of 3–5 cm in diameter [11]. By analyzing 2 RCTs and 5 cohort studies, Akkermann et al. found that the overall rate of DGE was significantly lower in patients who underwent gastric tube reconstruction compared with that in patients who underwent reconstruction using the whole stomach [9].

In 2002, Urschel et al. performed a meta-analysis including nine randomized controlled trials with a total of 553 patients [21]. According to this study, pyloric drainage procedures at the time of esophagectomy reduced the occurrence of early gastric outlet obstruction (*p* = 0.046) but had little effect on mortality, pulmonary complications and late postoperative foregut function.

Although two recent systematic reviews including 827 and 668 patients did not find a benefit of pyloric drainage procedures versus no intervention [9, 15], Arya et al. found a non-significant trend toward fewer anastomotic leaks, fewer pulmonary complications and less gastric stasis when pyloric drainage procedures were performed [8]. However, most studies included in these systematic reviews were performed at a time when the morbidity and mortality rates of esophagectomy were generally much higher compared to today. For example, the study of Akkerman et al. included patients who underwent surgery in the 1980s and 1990s or even earlier [9]. As mentioned above, the postoperative outcomes of these patients are not comparable to those of current studies due to improvements in intensive care and relevant modifications of operative techniques, including minimally invasive approaches.

In a recent randomized clinical trial, Mohajeri et al. found that pyloromyotomy or pylorus buginage could not reduce the incidence of DGE after esophagectomy with gastric pull-up [24]. Moreover, there is evidence that patients who undergo pyloric drainage procedures may suffer from increased biliary reflux and dumping syndrome long term [25, 26]. Wang et al. analyzed 368 patients following esophagectomy with esophageal substitute and found a greater incidence of both of these undesirable outcomes in patients who underwent pyloroplasty [27].

The present study aimed to evaluate surgical outcomes following esophagectomy with gastric replacement with a special focus on gastric outlet obstruction in a large single-center series. In this series, clinical signs of DGE were observed in 16.5% of the cases, and pneumonia was observed in 27.1% of the cases, which is consistent with the current literature. Most authors report a DGE rate ranging around 15% [28, 29], and most centers describe comparable rates of postoperative pneumonia [4]. Since no pyloric drainage procedures were performed in the current study, we can conclude that these procedures are not necessary. Moreover, most of the patients with clinical signs of DGE were successfully treated with conservative therapy. Only a few patients required endoscopic balloon dilation (3.8%), and none of the patients with DGE required reoperation or underwent a so-called rescue pyloroplasty.

In the present series, postoperative endoscopy with exploration of the anastomosis was performed whenever a patient had any obvious clinical signs or unexplained increasing levels of inflammatory parameters in the blood, which explains the relatively high rate of postoperative endoscopies. The reason for this approach is based on our experience that in the case of an anastomotic leak, an early intervention, such as stenting or placement of an endoscopic vacuum sponge, is helpful to prevent a severe and prolonged postoperative course. Some of the patients with anastomotic leakage only showed small leaks without necrosis and mild clinical symptoms, which explains the low rate of re-operation for anastomotic leakage and the relatively low 30-day hospital morbidity of 2.9%.

There are limitations in the present study design. First, this was a single-center observational study without a control group. According to the department standard, none of the patients underwent pyloric drainage procedures. Therefore, the data can only be compared to the existing literature. Still, we believe that our data are clinically relevant since many recent studies refer to historical clinical data of patients who underwent surgery in the 1980s or 1990s. Since this time, not only the surgical procedure but also postoperative intensive care management, including mobilization and nutrition regimens, have changed tremendously.

It has been reported that pyloric drainage procedures are not only ineffective in preventing DGE but also can add relevant morbidity, such as esophageal leakage or stenosis, in the long-term follow-up. For example, Richardson et al. described a patient who developed late pyloroplasty leakage on postoperative day 16 following esophagectomy [30]. Likewise, Antonoff et al. described two major complications directly related to pyloric drainage procedures, accounting for 0.6% of their study collective. One patient was re-explored on the first postoperative day because of bilious drainage from the midline abdominal incision due to a pinpoint hole at the pyloromyotomy. Another patient developed a leak at the pyloroplasty site and ultimately died following a complicated postoperative course [25]. Zieren et al. reported one patient who died following insufficient pyloroplasty and another patient who developed a severe stricture secondary to surgical pyloric drainage, accounting for 3.8% of their study group [31]. Although the overall surgical complication rate after pylorus drainage procedures seems to be relatively low, these practices are associated with significant morbidity and even mortality.

In more recent studies, the value of botulinum toxin injection in reducing gastric outlet obstruction was evaluated. In 2016, Fuchs et al. reported a trial in which 14 patients received botulinum toxin injections versus 27 who did not receive injections [32]. In this study, the rate of postoperative pyloric dysfunction was found to be significantly lower in the botulinum toxin group. Moreover, patients who received botulinum toxin injections were discharged earlier (7.4 versus 10.7 days, *p* < 0.05), and no differences were observed regarding anastomotic strictures or leaks. In contrast, Eldaif et al. found that patients receiving botulinum injections exhibited a higher rate of postoperative reflux symptoms and increased use of promotility agents and more frequently required postoperative endoscopic interventions. Therefore, the authors concluded that intrapyloric botulinum toxin injections should not be used as an alternative to standard drainage procedures [33]. Consequently, the value of botulinum toxin injections as an alternative approach to reduce postoperative gastric outlet obstruction remains controversial. More randomized clinical studies with larger samples are required before this method can be generally recommended.

For postoperative DGE therapy, conservative approaches or endoscopic pyloric balloon dilation are safe and effective in most patients [34–36]. For example, Maus et al. performed 89 pylorus balloon dilations after esophagectomy without complications. In this study, the total re-dilation rate for a 30-mm balloon was 20% [37]. In rare cases of endoscopic therapy failures, rescue pyloroplasty has been described to be helpful and well tolerated. For example, Datta et al. reported that rescue pyloroplasty was successful in 9 of 13 cases (69%), leading to decreased rates of nausea, vomiting, bloating, prokinetic use, and total parenteral nutrition dependence [38].

## Conclusions

Our current single-center study aimed to evaluate whether pyloric drainage procedures are necessary to achieve an acceptable early postoperative outcome. In the present series, DGE was observed in the early postoperative course in 16.5% of cases. Pneumonia as a potential consequence of gastric outlet obstruction was observed in 27.1% of cases. Both of these values are consistent with the current literature regardless of whether pyloric drainage procedures were applied. Therefore, we propose that pyloric drainage procedures may be unwarranted in thoracoabdominal esophagectomy. In fact, these procedures can be associated with complications that may lead to morbidity or even mortality. To ultimately address the question of whether pylorus drainage procedures are beneficial versus no intervention, future prospective randomized controlled trials are needed.

## Abbreviations

ASA: American Society of Anesthesiologists
BMI: Body mass index
DGE: Delayed gastric emptying
EUS: Endoscopic ultrasonography
GIST: Gastrointestinal stroma tumor
MRI: Magnetic resonance imaging
NET: Neuroendocrine tumor
